# What are the personal last wishes of people with a life-limiting illness? Findings from a longitudinal observational study in specialist palliative care

**DOI:** 10.1186/s12904-022-00928-1

**Published:** 2022-03-22

**Authors:** Anneke Ullrich, Wiebke Hollburg, Holger Schulz, Sven Goldbach, Annette Rommel, Marten Müller, Denise Kirsch, Katrin Kopplin-Foertsch, Julia Messerer, Louise König, Frank Schulz-Kindermann, Carsten Bokemeyer, Karin Oechsle

**Affiliations:** 1 Palliative Care Unit, Department of Oncology, Hematology and BMT, University Medical Center Hamburg-Eppendorf, 20246 Hamburg, Germany; 2 Specialist Outpatient Palliative Care Team “PalliativPartner Hamburg GbR”, Hamburg, Germany; 3 Department of Medical Psychology, Center for Psychosocial Medicine, University Medical Center Hamburg-Eppendorf, Hamburg, Germany; 4 Specialist Outpatient Palliative Care Team „Das Palliativteam“, Hamburg, Germany; 5 Palliative Care Ward, Asklepios Hospital Rissen, Hamburg, Germany; 6 Specialist Outpatient Palliative Care Team Hamburg-West, Hamburg, Germany; 7 Palliative Care Ward, Agaplesion Diakonie Hospital, Hamburg, Germany

**Keywords:** Palliative Care, End-of-life Care, Patient, Wish, Patient-centered care, Longitudinal Study

## Abstract

**Background:**

Personal last wishes of people facing a life-limiting illness may change closer to death and may vary across different forms of specialist palliative care (SPC).

**Aims:**

To explore the presence and common themes of last wishes over time and according to the SPC settings (inpatient vs. home-based SPC), and to identify factors associated to having a last wish.

**Methods:**

Patients enrolled in a longitudinal study completed questionnaires at the onset (baseline, t_0_) and within the first 6 weeks (follow-up, t_1_) of SPC including an open-ended question on their personal last wishes. Last wishes were content analyzed, and all  wishes were coded for presence or absence of each of the identified themes. Changes of last wishes (t_0_-t_1_) were analyzed by a McNemar test. The chi-square-test was used to compare the two SPC settings. Predictors for the presence of a last wish were identified by logistic regression analysis.

**Results:**

Three hundred sixty-one patients (mean age, 69.5 years; 49% female) answered at t_0_, and 130 at t_1_. In cross-sectional analyses, the presence of last wishes was higher at t_0_ (67%) than at t_1_ (59%). Comparisons revealed a higher presence of last wishes among inpatients than those in home-based SPC at t_0_ (78% vs. 62%; *p* = .002), but not at t_1_. Inpatient SPC (*OR* = 1.987, *p* = .011) and greater physical symptom burden over the past week (*OR* = 1.168, *p* < .001) predicted presence of a last wish at t_0_. Common themes of last wishes were *Travel*, *Activities*, *Regaining health*, *Quality of life*, *Being with family and friends*, *Dying comfortably*, *Turn back time*, and *Taking care of final matters*. The most frequent theme was *Travel*, at both t_0_ (31%) and t_1_ (39%). Themes did not differ between SPC settings, neither at t_0_ nor at t_1_. Longitudinal analyses (t_0_-t_1_) showed no significant intra-personal changes in the presence or any themes of last wishes over time.

**Conclusions:**

In this late phase of their illness, many patients voiced last wishes. Our study suggests working with such wishes as a framework for person-centered care. Comparisons of SPC settings indicate that individualized approaches to patients’ last wishes, rather than setting-specific approaches, may be important.

## Background

Death can be seen as a transition process and is inevitable and irreversible among human lives [1]. Depending on the individual’s perspective, a “good death” may be defined by different attributes, such as awareness or acceptance of the end of life, relief from suffering and pain, having hope, dignity, presence of family, and good communication [2–4]. Studies show that most seriously ill and dying people see general importance in preparing for death [5–7]. To conceive bucket lists [8] or to have a special wish come true can be interventions that focus on preparing for anticipated death.

The main aim of palliative care is to improve the quality of life by preventing or relieving suffering in all domains of human suffering with respect to symptom control, psychosocial and spiritual support and practical issues [9]. Given the underlying holistic approach, it is vital to explore personal last wishes as part of routine palliative care. A personal last wish in palliative care can be characterized as a felt or expressed desire not limited to the medical domain, which, to the affected patient, is personally valuable in his/her final phase of life. So far, however, palliative care research has rather focused on end-of-life care goals than personal last wishes. Although end-of-life care goals among seriously ill patients were found to reveal more profound wishes and desires beyond physical needs, [10] the framing of such  goals predominantly occurs in the medical context aiming to “harmonize patient’s treatment choices with their values and medical conditions” [11]. Seriously ill and dying people want to be helped “living a meaningful life”, [12] which may include the desire to talk about and to accomplish personal last wishes beyond the medical context. Likewise, what matters most for palliative care from the perspective of healthcare professionals includes the fulfillment of patient’s wishes [13]. Knowledge about patients’ personal last wishes may help healthcare professionals provide individualized, compassionate care and support patients’ notion of a meaningful life. However, healthcare professionals may find it challenging to identify a patient’s personal last wishes at the end of life, especially when death is nearing and the most complex physical and emotional problems arise [14].

Although personal last wishes are unique to every person, they may revolve around similar topics. Previous studies in people facing a life-limiting illness and community samples identified different themes reflecting wishes before death. These involved the areas of family, activities, life fulfillment, love and happiness, the greater good, peace, religion, legacy, gratitude, and health [15–18]. Among advanced cancer patients, the most frequent wishes were being free from pain, not being a burden to the family, saying goodbye, and being at peace with god [8]. It has also been shown that many patients express a desire to return to their place of birth at the end of life [19]. Actual listings of personal last wishes and their frequency have been rarely investigated among seriously ill and dying people receiving specialist palliative care (SPC). However, increased awareness for quality of life issues and psychosocial interventions during SPC may raise personal last wishes of patients or prompt verbalizing specific wishes. Further, it is assumed that wishes and priorities of people facing a life-limiting illness may change closer to death [16]. Thus, eliciting and dealing with personal last wishes is highly relevant in the context of SPC, as referral to SPC still occurs late in the illness trajectory, sometimes only days before death [20].

Little is known about possible variations of patient’s personal last wishes depending on the SPC setting. Nonetheless, setting-specific information about the nature of such wishes may improve healthcare professionals’ ability to work with such wishes. In Germany, similar to worldwide structures, SPC is provided by specialized services for patients with complex and difficult problems and needs that cannot be adequately covered by other treatment options [21, 22]. There are different care settings where SPC services could be delivered, including home-based and inpatient SPC. Home-based SPC is most often based on bi-professional care provided by specialized physicians and nurses. In the home care setting, services of other professions in the psycho-social field are not covered by health insurance so that cooperation with professionals specialized in spiritual care, social work, and psychology is infrequent [23]. In contrast, inpatient SPC is delivered by multi-professional teams, in which psychologists, social workers and spiritual workers with recognized specialist training are an integral part. Due to more psychosocial conversation offers, inpatients may recognize their personal last wishes more intensely compared to patients referred to home-based SPC. Further, indications for inpatient SPC are complex symptomatology, uncertainty in setting treatment goals, complex medical or nursing care, and overstrained home care [24]. Differences in themes of personal last wishes may occur due to an increased awareness of limited resources and time left for wish fulfilment (i.e., limited functioning, hospitalization).

Taken together, little is known about personal last wishes in the context of SPC. Consequently, this longitudinal exploratory study seeks to investigate the presence and common themes of personal last wishes, as well as how these wishes may change over time among patients receiving SPC. Further study aims were to identify factors associated with the presence of a personal last wish at the onset of SPC as well as to examine how such wishes may vary depending on the SPC setting to which the patient was referred.

## Materials and methods

### Design and setting/context

The data come from a longitudinal observational multi-center study designed to investigate personal last wishes, psychological burden, and unmet needs of people with life-limiting illness in the course of home-based and inpatient SPC. The present exploratory analysis focuses on personal last wishes at the onset and during SPC, based on free-text responses from questionnaires; results on the other study outcomes will be published separately [25].

Between June 2017 and July 2018, participants were consecutively enrolled in six study sites, each including three home-based and inpatient SPC services of an urban SPC network in Hamburg, Germany.

### Eligibility criteria

Inclusion criteria were (1) being over the age of 18, (2) presence of an advanced, life-limiting disease (cancer and non-cancer), and (3) entering home-based or inpatient SPC for the first time. Exclusion criteria were (1) cognitive impairment or language problems interfering with giving informed consent and/or answering questionnaires, (2) acute physical or psychological crisis so that study participation would impose relevant burden, and (3) imminent death (as assessed and judged by staff members). Inclusion/exclusion criteria were included in a screening script for staff members. Reasons for study exclusion or non-participation were systematically documented by means of a standardized form.

### Procedures

Staff members of the six SPC services consecutively identified eligible patients and approached potential participants within the first 72 h of SPC, which we will call the onset of SPC. Staff members then asked consenting patients to complete a self-report questionnaire, which included a study-specific open-ended question on personal last wishes, as well as standardized, validated measures on psychological and physical patient outcomes. The interdisciplinary research team, who developed the questionnaire in consultation with a reference group of representatives of the involved SPC services, consisted of palliative care experts with professional backgrounds in medicine, nursing, psychology, and sociology.

If requested, staff members could assist the patient in writing down the responses. On the last page of the questionnaire, patients were asked to state whether they completed the questionnaire with or without external help. To ensure confidentiality, patients returned their filled questionnaires in a sealed envelope.

Each participant completed the same questionnaire at two time points: At first within 72  h of SPC (baseline, t_0_), and at follow-up between 1 to 6 weeks after the onset of SPC (t_1_). In the follow-up period, participants either completed a questionnaire when being referred to any other healthcare facility or after four weeks of care in the same SPC service. The first questionnaire returned during the follow-up period served as follow-up data. Further assessments were conducted according to a follow-up schedule but due to limited sample sizes, those were not included in the current analysis.

### Measures for maintaining data integrity

In order to maintain study quality, each participating service determined at least one responsible person for equipping staff members with study material and managing data collection at follow-up. Staff members involved in the conduct of the study (primarily physicians and nurses) were trained in central workshops (AU, HS, FSK) to standardize study procedures and documentation. In this context, staff members were also familiarized with inclusion/exclusion criteria, thus they adhered to them when recruiting participants. At half the recruitment period follow-up workshops were held to allow staff members to share their experiences and ensure study adherence.

### Ethics

The ethics committee of the General Medical Council of Hamburg, Germany, approved the study (reference number PV5062). The study was conducted according to the principles expressed in the Declaration of Helsinki and guidelines to accommodate ethical issues in research on palliative care [26]. All participants provided written informed consent before taking part in the study.

## Measurements

### Personal last wishes

The presence of a personal last wish and themes of such wishes at the onset of SPC (t_0_) and at 1 to 6-weeks follow-up (t_1_) represented the primary outcomes in our analyses.

The presence or absence of such a wish was assessed with the following study-specific question: *“Do you have a personal last wish? (answering options: yes/no). If you answered “yes,” please provide a description of your personal last wish.”* Thus, following the closed question format on the presence of a personal last wish, descriptions of these wishes were prompted by an open-ended question.

The question was empirically formulated based on consensus views of those who were part of the questionnaire design process. The term “personal last wish” was not further defined for participants.

### Potential predictor variables

We examined the following comprehensive set of psychological, physical, socio-demographic, and disease-/care-related variables that may be associated with the presence of a personal last wish at the onset of SPC (t_0_). Potential factors were identified based on the clinical experiences of the interdisciplinary research team.

The German version of the Distress Thermometer (DT) [27] was used to assess psychological distress over the past week on an 11-point analog scale. A cut-off value of ≥ 5 reflects clinically relevant distress with the need for professional psychosocial support. The DT is accompanied by a list of potentially distress-causing problems, including 21 distinct physical problem items (i.e., pain, dyspnea, fatigue, sleep). Patients reported whether they were bothered by any of these problems rated 0 (no) and 1 (yes). We used the physical symptom count (0–21) as an indicator of physical symptom burden.

Depression and anxiety over the past two weeks were measured by the 4-item Patient Health Questionnaire (PHQ-4, German version), [28] consisting of a two-item anxiety scale (GAD-2) and a two-item depression scale (PHQ-2). GAD-2 and PHQ-2 sum scores each range from 0 to 6, with higher values representing higher symptom levels, and scores of ≥ 3 indicate suspected anxiety disorder or depression [29, 30]. We used the sum scores to estimate the patient’s levels of anxiety and depressive symptoms.

Further, patients reported about socio-demographic characteristics (i.e., age, marital status, educational level), disease-related data (i.e., primary disease), and care-related data (i.e., SPC setting, advanced directives, nursing situation before the onset of SPC). To evaluate the nursing situation, information about the level of informal and institutional care before the onset of SPC was obtained (no nursing, by relatives only, nursing service only, nursing service and relatives).

## Data Analyses

### Qualitative data analysis

A content analysis approach following Mayring [31] was applied to inductively analyze qualitative responses of patients reporting a personal last wish at the onset of SPC (t_0_) and/or follow-up (t_1_). Qualitative content analysis is suitable for identifying common issues mentioned in data [32] and measuring the frequency of different categories [33].

A researcher experienced in qualitative research (AU, sociologist), who was not involved in the provision of SPC, first categorized the descriptions of patient’s personal wishes. Presentations of wishes mainly consisted of keywords or small phrases. Different levels of categories (main categories and subcategories) were identified and regularly discussed within the research team. A second rater (LK, psychologist) then used the coding scheme created from this process and independently categorized the responses. Each personal last wish was only added to one category. In case of mismatching categorization, discussions between the two researchers were pursued until consensus was reached. MaxQDA12 facilitated managing and analyzing qualitative data. The Consolidated Criteria for Reporting Qualitative Research (COREQ) guidelines were applied to report results [34].

### Quantitative data analysis

Themes of personal last whishes at the onset of SPC (t_0_) and follow-up (t_1_) were coded for the presence or absence of each of the categories by assigning a value of 0 (no) or 1 (yes). Thus, frequencies were calculated on patient-level as percentages of patients who reported at least one personal last wish referring to the respective category.

Cross-sectional group differences between personal last wishes of patients receiving home-based vs. inpatient SPC were analyzed by chi-square-tests, concerning both the presence and themes of personal wishes.

Intra-personal changes (t_0_-t_1_) in both the presence and themes of personal last wishes were investigated by reporting proportions of patients who newly reported, no longer reported, and steadily reported/did not report a wish. Exact McNemar test for related samples was used to assess statistical significance. Due to the exploratory approach of the comparisons abovementioned, no adjustment for multiple testing was performed [35, 36].

In order to identify predictors for the presence of a personal last wish at the onset of SPC (t_0_), multivariable binary logistic regression analysis was used. Socio-demographic variables (age, gender, marital status, having children, living alone, religious confession, and education), disease-/care-related variables (primary disease, physical symptom count, advanced directives, and SPC setting) and psychological variables (scores of distress, anxiety symptoms, and depressive symptoms) were considered as potential predictors. Potential predictor variables were entered simultaneously into the multivariable model (method: enter). Missing data were handled by list-wise deletion, resulting in a final sample of 349 out of 361 (97%) patients. Strengths of associations were expressed as odds ratios (*OR*) with 95% confidence intervals (CI). To evaluate the goodness-of-fit of the logistic model, we used Nagelkerke’s pseudo R^2^ [37] with values > 0.2 being considered acceptable [38].

Analyses were performed using SPSS software version 25 (IBM, USA). All significance tests were two-tailed using a significance level of *α* < 0.05.

## Results

### Participants

**Fig. 1:**
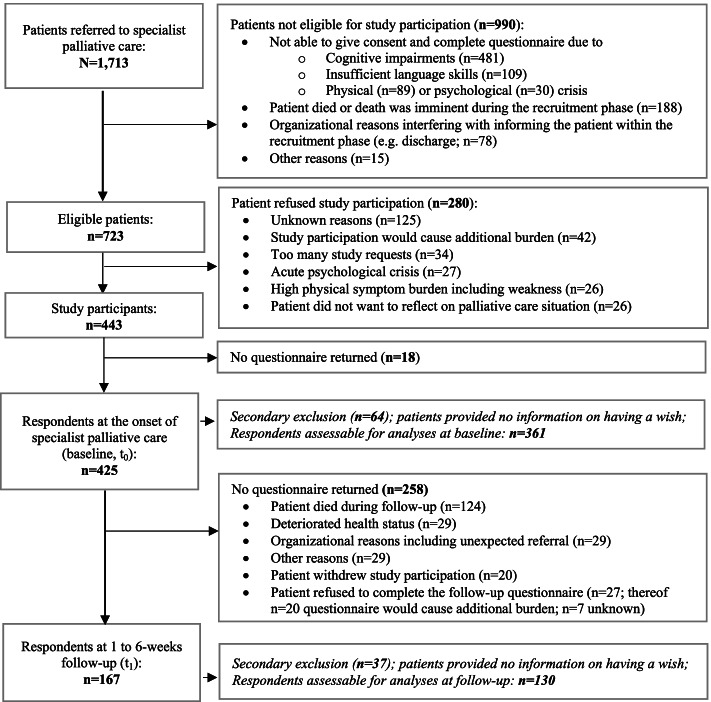
Flow diagram for the creation of the study sample

The flow diagram for the creation of the study sample is displayed in Fig. 1. In the recruitment period, 990 (58%) of 1,713 patients had to be excluded. SPC settings differed significantly in the proportions of those excluded due to imminent death (home-based SPC: 20%, inpatient SPC: 80% of 188; *p* < 0.001), organizational reasons (26% vs. 74% of 78; *p* = 0.007), cognitive impairments (44% vs. 56% of 481; *p* = 0.028), and insufficient knowledge of German (61% vs. 39% of 109; *p* < 0.001). Of the remaining 723 patients, 280 (39%) declined study participation. Regarding the two SPC settings, the proportions of patients who refused to participate did not differ significantly (home-based SPC: 51%, inpatient SPC: 49% of 280; *p* = 0.202).

Finally, 443 patients were enrolled in the study, of which 425 (59% of those eligible) delivered a questionnaire at the onset of SPC (t_0_). However, cross-sectional analyses were restricted to 361 participants (85%) who responded to the question regarding personal last wishes. Item-responders and non-responders did not differ regarding age (*p* = 0.497), gender (*p* = 0.193), primary disease (*p* = 0.158) and SPC setting (*p* = 0.239).

During the follow-up period, 124 (29%) of 425 patients died. Of the remaining 301 patients, 167 (55%) returned a questionnaire at 1 to 6-weeks follow-up (t_1_). Cross-sectional analyses were restricted to 130 participants (78%) who responded to the question regarding personal last wishes. Again, item-responders and non-responders did not differ regarding age (*p* = 0.288), gender (*p* = 0.193), primary disease (*p* = 0.158) and SPC setting (*p* = 0.647).

Overall, 114 (88%) of 130 participants provided outcome data at both points of time, representing the sample for exploratory longitudinal analyses (t_0_-t_1_).

Characteristics of the 361 participants according to the timing of the onset of SPC (t_0_) are presented in Table 1. Of these participants, 52% were male, and 91% were cancer patients. On average, participants were 69.5 (SD 12.5) years of age. Two-thirds (67%) were initially referred to home-based SPC; those participants were on average six years older (*p* < 0.001) and reported slightly higher levels of anxiety symptoms (*p* = 0.045) compared to those initially referred to inpatient SPC.

At 1 to 6-weeks follow-up (mean 3.6 weeks after t_0_, SD 1.4; median 4.0), 73% of the remaining 130 participants were still treated by the same SPC service to which they had been initially referred, while 27% had changed the care setting (i.e., home-based to inpatient SPC and vice versa). At this point, three-quarters (76%) of the 130 remaining participants were cared for by home-based SPC.

## Themes of personal last wishes

Table 2 shows the categories, subcategories and example quotes of personal last wishes. Qualitative content analysis revealed eight categories of specific themes of personal last wishes and a ninth category related to the meaning of such wishes. The eight themes of personal last wishes were *Travel*, *Activities*, *Regaining health*, *Quality of life*, *Being with family and friends*, *Dying comfortably*, *Turn back time*, and *Taking care of final matters*.

Analyses revealed insights into the features of personal last wishes. For example, wishes belonging to the category of *Travel* were often interlinked with notions on family and friends, and implicated the aspect of spending time together (i.e., “to go to Denmark, and relax in our cabin with my wife”). Family members were also mentioned in other categories, such as *Taking care of final matters* (i.e., “that the kids will make peace with each other”) or *Activities* (i.e., “to renovate the house and to enjoy my father’s astonishment”). Personal last wishes attributed to *Travel* and pleasant *Activities* often included a component of reconnection with earlier times and the old self (i.e., vacation in a well-known place, visit/return to place of birth, activities the patient used to do). Few wishes were too global or vague to attribute to a specific category (i.e., “a wish stays a wish”), which were not included in further analyses.

 An additional category referred to the meaning of personal last wishes. Some wishes represented goals of care, life goals, or experiences that patients wanted to have before dying, indicating a desire for fulfillment and realization. In contrast, some wishes voiced aspects of hope or dreams/fantasies. For the latter, illustrating examples from patient’s written wishes are “I hope for a cure,” “I am waiting for the miracle to be healthy,” and “I’ve always dreamt of stroking leopards”.

## Presence/absence of a personal last wish and themes of personal last wishes

### Cross-sectional analysis at the onset of SPC

Table 3 presents data on personal last wishes among 361 patients at the onset of SPC (t_0_), of which 243 (67%) reported the presence of a personal last wish. *Travel* (31%), *Activities* (18%), and *Regaining health* (40%) were the three most frequent themes of personal last wishes among these patients. Comparison between groups revealed that the presence of a personal last wish was significantly lower in home-based SPC compared to inpatient SPC. However, there was no difference between groups on any themes of personal last wishes.

### Cross-sectional analysis at follow-up

As shown in Table 4, 77 (59%) patients reported presence of a personal last wish at 1 to 6-weeks follow-up (t_1_). The three most frequent themes of these wishes were *Travel* (39%), *Regaining health* (22%) and *Quality of life* (20%). Opposed to the onset of SPC (t_0_), no personal last wishes in the category of *Turn back time* were mentioned at follow-up. Frequencies of personal wishes did not differ between home-based and inpatient SPC, neither in their presence nor in any themes of wishes. 

## Exploratory longitudinal analysis of personal last wishes

Intra-personal changes of personal last wishes over time were assessed by comparing data collected at the onset of SPC and at 1 to 6-weeks follow-up in a subgroup of 114 patients (t_0_-t_1;_ Table 5). Of these, 53 patients (47%) steadily reported having a personal last wish, while each 15–20% were categorized as newly reporting, no longer reporting, or steadily not reporting a personal last wish. However, these changes were not statistically significant. Additionally, there were no significant intra-personal changes of any themes of personal last wishes over time.

## Factors associated with the presence of a personal last wish at the onset of specialist palliative care

Univariable regression analyses (Table 6) showed that the following variables were not associated with the presence of a personal last wish at the onset of SPC (t_0_): age, gender, marital status, having children, living environment, religious denomination, primary disease, advanced directives, levels of distress, and depressive symptom levels. Thus, these factors were dropped from the model, whereas education, SPC setting, physical symptom count, and anxiety symptom level were included in multivariable analysis. The remaining variables were tested for multicollinearity, but each showed variance inflation factors of less than 1.4. Hence, multicollinearity did not pose a problem [39].

Results of multivariable regression analysis (Table 7) showed that only initial referral to inpatient SPC (vs. home-based SPC; *OR* = 1.987, *p* = 0.011) and a higher physical symptom burden over the past week (*OR* = 1.168, *p* < 0.001) were significantly associated with the presence of a personal last wish.

## Discussion

This study aimed to examine the presence and themes of personal last wishes among patients at the onset and during SPC, as well as factors associated with having a wish. Further, patients receiving home-based and inpatient SPC were compared.

Four major findings emerged. First, personal last wishes are frequent in patients at the onset of SPC and were found to be associated with initial referral to inpatient SPC and a higher physical symptom burden over the past week. Second, both the presence of a personal last wish and themes of such wishes appear to be relatively stable within the first 6 weeks of SPC. Third, patients receiving home-based and inpatient SPC do not differ significantly regarding themes of personal last wishes, but – at least at the onset of SPC – a greater proportion of inpatients reported the presence of a personal last wish. Lastly, different themes of personal last wishes could be identified in the patient’s responses, shedding light on the nature and meaning of personal last wishes in the context of SPC.

## Presence of personal last wishes and associated factors at the onset of SPC

Over two-thirds of patients reported the presence of a personal last wish at the onset of SPC, suggesting that such wishes are common among this patient group. For identification of key factors associated with reporting a wish, we considered a range of variables, including socio-demographic, disease-/care-related, and psychological variables. Multivariable logistic regression analysis revealed initial referral to inpatient SPC and greater physical symptom burden to be the only significant predictors. Thus, patients with a mounting number of physical symptoms should be evaluated closely for having a personal last wish. Yet it is essential to be mindful that the physical symptom burden as captured in our study does not provide information on symptom severity. Possibly, a patient could have only one severe symptom impairing his/her functioning, while another one might have several symptoms with no such impairments. Interestingly, the presence or absence of a personal last wish does not seem to be associated with indicators of worse psychological adjustment, such as elevated levels of anxiety or depressive symptoms.

## Changes of the presence and themes of personal last wishes

Cross-sectional analysis revealed that the presence of a personal last wish at follow-up decreased compared to the onset of SPC, but remained at well over 50% of patients. Our exploratory longitudinal analysis in of a subgroup of patients showed that about two-thirds persistently reported either having a personal last wish or not, whereas in the other third having a wish changed over time. However, our study cannot elucidate the reasons behind the variability of having a wish in these patients (i.e., because the wish was fulfilled or because patients lost their hope) due to the lack of assessment about why personal last wishes changed.

## Comparison of the presence and themes of personal last wishes between SPC settings

Presence of personal last wishes varied by SPC setting, with a greater proportion of inpatients compared to those in the home-based setting having such a wish at the onset of SPC. It might be that patients associate inpatient SPC with imminent death at first [40], and their perceptions might catalyze cognitive processes regarding personal last wishes. It is also possible that better acceptance of the end-of-life situation, which seems related to expressing a wish [16], might be more prominent in inpatient SPC and prompts patients to verbalize their wishes. However, we found no significant differences on the presence of personal last wishes at follow-up. Moreover, themes of personal last wishes did not vary between SPC settings. These findings indicate that individualized approaches to patients’ last wishes, rather than setting-specific approaches, may be relevant in the late phase of a life-limiting illness.

## Identification of themes of personal last wishes

We identified eight themes of specific wishes: *Travel*, *Activities*, *Regaining health*, *Quality of life*, *Being with family and friends*, *Dying comfortably*, *Turn back time*, and *Taking care of final matters*. Most themes are in line with studies that have investigated wishes, bucket lists, or important values among patients with a life-limiting illness [8, 16, 18] as well as community views [15, 41]. Nonetheless, in our study, *Turn back time* was found to be a theme not reported in prior studies. Confronted with the finitness of life, patients expressed that they wished to go back in time, for example to be young again or have a time machine to go back in time and change things. Given the method of data collection of our study, richness of the underlying data is limited and interviews were needed to gain deeper insights into wishes regarding *Turn back time*.

The most frequent personal last wishes reported in our study were associated with *Travel* and *Activities*. Such wishes often included reconnection with the old self, for example travel destinations that had a special meaning in the patient’s life. In line with these findings, a study from the U.S. also showed that patients with life-limiting diseases often express travel desires, including returning to the place of birth to die [19]. Additionally, in a survey with nearly 3,000 people across the U.S. (community sample), travel was the most prevalent theme on bucket lists (79%) [41]. It is important to note that these findings might be culturally bound. Personal wishes relating to raveling and activities might be especially prominent in Western culture.

With regard to final matters, finding forgiveness and reconciliation is an essential clinical theme in the process of dying [42]. Unfinished business at the end of life can relate to family-related and responsibilities, relationships, personal goals, professional work, and the organization of home [43]. Our analyses, in turn, revealed that only a few patients reported personal last wishes regarding *Taking care of final matters*. This might be a methodologically influenced finding since these are intimate questions people might find painful to address. For some, it might not be easy to report through written prompts but might reach better through face-to-face interviews.

Beyond the identification of specific themes of personal last wishes, our results pointed to different meanings of such wishes: they may represent goals or experiences for which the patient desires fulfillment, but they also may voice a hope or a dream/fantasy. A sensitive exploration of these meanings can be a therapeutic opportunity for a compassionate dialogue with the ill person and his/her family. Beneficial aspects could include the following: At first, plans for how a specific wish may be fulfilled can be discussed. If fulfillment seems unrealistic due to the patient’s condition or other reasons, healthcare professionals can help patients to identify new wishes, resulting in a reframing of hope [44]. Second, asking for wishes and their meaning could be used to introduce conversations about the end of life and death. Third, if a wish rather voices hopes or dreams/fantasies, room can be given for discussions about fond memories, appreciation of the enclosed hope and vibrancy, or aiding the life-review process and biography work. Frameworks and approaches to these discussions have been described with regard to conversations about hope [45] and life-review [46].

The last months and weeks of life represent an important time for seriously ill and dying patients and their families. Conversations about wishes and priorities allow patients to define their preferences for dying and death [47]. Previous research [42, 48] emphasized that patients’ wishes should be considered when consulting on treatment options, care, and time planning. In our study, some themes of personal last wishes directly related to the medical context, quality of life and the dying process. However, most commonly reported personal last wishes related to a wider context of life such as travel, activities, and relationships. Therefore, being informed about patients’ personal last wishes can enhance healthcare professionals’ understanding of what matters most to patients, [41] assuring holistic and compassionate care when life comes to an end. Communication about end-of-life issues is most challenging, and various skills and strategies are required for meaningful and effective conversations about patient’s desired care, [49] including personal last wishes. Different tools have been developed and tested to facilitate conversations about wishes, preferences, and end-of-life care goals between healthcare professionals and patients [16, 50]. For example, in one study 46 statement cards on possible wishes and priorities were developed that helped raise awareness and verbalize what matters most to the patient [16]. However, the authors considered the number of cards as too large for the illest and frail participants, [16] which may affect its practicability in SPC. Findings from the present study might guide as to which of the cards may be most relevant for patients in need of SPC.

Lastly, it is important to acknowledge that death and dying are intertwined with sociocultural values and beliefs [51]. Underlying assumptions about a “good death” and the healthcare professionals’ role in supporting conversations about patient’s personal last wishes come from a Western model, which places a high value on death awareness [52]. It is essential to be mindful about the philosophical framework that underpins the palliative care paradigm, since cultural perceptions might influence patients’ preferences to engage in a conversation with healthcare professionals about their personal last wishes. In consequence, healthcare professionals are required to identify and respect the particular nuances of cultures from which their patients come.

## Strengths and limitations

The study addresses a gap in knowledge by answering a research question with personal last wishes of seriously ill and dying people receiving SPC. Strengths of the study include the longitudinal design, the multi-center approach including inpatient and home-based SPC, consecutive recruitment strategy, and the systematic documentation of non-participants and non-responders. The mixed methods used to analyze the data add depth to understanding the topic of personal last wishes.

However, some limitations should be noted. First, the generalizability of the results may be limited as more than half of patients referred to SPC were ineligible for study participation, many of them due to proximity to death and cognitive deficits. In addition, given that nearly half died before completing the second questionnaire, the sample predominantly consisted of people in the last weeks of their lives. Further, over 90% of participants had cancer. In Germany, the majority of patients in SPC services are cancer patients [53]. Yet, given that SPC’s embracing approach includes all people with a life-limiting disease regardless of diagnosis or prognosis, this can be another limitation.

Second, results have to be interpreted with caution since sample sizes at follow-up were small, and the sample size and power of longitudinal data had not been determined. Longitudinal assessments were limited by deteriorated health conditions and refusal to continue answering questionnaires. These are well-known problems in palliative care research, [54] since patient’s needs and health condition have to be accommodated due to ethical reasons [26, 55].

Other problems concern the substantial number of patients that did not answer the question on personal last wishes. In addition, we have to acknowledge the possibility that patients answered that they did not have any last wishes, but in reality did. Possible reasons might include lack of motivation, diminished energy or capacity to concentrate the question, or not recognizing desires as “personal last wishes”. Yet a majority responded to the question, which should be assuring and motivating healthcare professionals to elicit patients’ personal last wishes.

Further, approaching personal last wishes through a questionnaire can be questioned. Written reports of such wishes instead of interviews might be difficult for patients receiving palliative care, and we cannot interpret what lies behind the patients’ silence. Moreover, it does not allow collecting context information (i.e., biographical, social, spiritual, or moral understandings) that would give insights into how patients understand their statements.

## Conclusions

We explored personal last wishes of patients at the onset of SPC and in the first weeks of care. Findings on such wishes can help improve decision-making processes in palliative care and generally support more compassionate, individualized care for the seriously ill and dying. Our study supports the exploration of personal last wishes, but future research on this topic is warranted. Further research should explore personal last wishes through face-to-face interviews with patients and find out how these are connected to inherent personal values and meaning. Deeper insights into patient’s personal last wishes could help to clarify how healthcare professionals can explore these wishes together with the patients. As family caregivers were often mentioned in patients’ wishes, it would also be interesting to know their perspective to contextualize these wishes. Future research could include patient-caregiver dyad’s communication on personal last wishes, family caregivers’ attending to these wishes or dealing with unrealistic wishes, and own needs associated with the patient’s wishes.

**Table 1:** Characteristics of the study sample at the onset of specialist palliative care

		**Entire sample** **(*N* = 361)**		**Home-based SPC** **(*N* = 238)**		**Inpatient SPC** **(*N* = 123)**		
		n	%	n	%	n	%	p
**Sociodemographic factors**								
Age (M, SD)		69.5 (12.5)		71.5 (10.9)		65.6 (14.4)		** < .001**
Gender	Female	178	49.4	119	50.2	64	52.0	.739
	Male	182	50.6	118	49.8	59	58.0	
Religious confession	Yes	202	57.7	126	55.3	76	62.3	.204
Family status	Single	67	18.6	37	15.6	30	24.4	.104
	Married or life partnership	178	49.4	119	50.2	59	48.0	
	Divorced or widowed	115	31.9	81	34.2	34	27.6	
Have children	Yes	258	71.9	170	71.7	88	72.1	.936
Living environment	Living alone	119	33.5	81	34.9	38	30.9	.445
	Living in the same household or near the family	236	66.5	151	65.1	85	69.1	
School education^a^	Low (≤ 9 years)	143	40.5	96	41.6	47	38.5	.679
	Intermediate (10 years)	99	28.0	66	28.6	33	27.0	
	High (12–13 years)	111	31.4	69	29.9	42	34.4	
**Disease-/care-related factors**								
Primary disease	Gastrointestinal cancer	83	23.0	45	18.9	28	30.9	.058
	Cancer of the respiratory system	66	18.3	50	21.0	16	13.0	
	Urogenital and breast cancer	115	31.9	78	32.8	37	30.1	
	Other malignancies	63	17.5	40	16.8	23	18.7	
	Not malignant	34	9.4	25	10.5	9	7.3	
Physical symptom count (0–21) (M, SD)		10.3 (3.6)		10.1 (3.7)		10.7 (3.3)		.137
Nursing situation before the onset of SPC^b^	No nursing	70	20.3	37	16.4	33	27.7	.054
	By relatives only	126	36.6	83	36.9	43	36.1	
	Nursing service only	101	29.4	74	32.9	27	22.7	
	Nursing service and relatives	47	13.7	31	13.8	16	13.4	
Advanced directives – living will	Yes	244	67.6	161	67.6	73	67.5	.974
Advanced directives – healthcare proxy	Yes	231	64.0	156	65.5	75	61.0	.391
**Psychological factors**								
Distress level (DT, 0-10) (M, SD)		7.2 (2.2)		7.1 (2.1)		7.3 (2.2)		.563
Anxiety symptom level (GAD-2, 0–6) (M, SD)		2.4 (2.0)		2.5 (2.0)		2.1 (2.0)		**.045**
Depressive symptom level (PHQ-2, 0–6) (M, SD)		3.0 (2.0)		3.0 (1.9)		3.0 (2.1)		.954

*Abbreviations*: *DT* Distress Thermometer, *GAD-7* Generalized Anxiety Disorder 2-item Scale, *M* mean; *p* probability of type I error (chi-square-test for ordinal variables, two-sample t-test for continuous variables), *PHQ-2* Patient Health Questionnaire – 2-item Depression Module, *SD* standard deviation, *SPC* specialist palliative care

Significant results are marked in bold

^a^Low: secondary general school-leaving certificate (leading to 3-year apprenticeship or to secondary vocational schools) or less, intermediate: intermediate school-leaving certificate (leading to 2–3 year apprenticeship, to secondary vocational, general schools or attaining a high school diploma, high: university/college entrance qualification; ^b^Need for informal and/or institutional care prior to being referred to SPC

**Table 2:** Themes of personal last wishes revealed by qualitative analysis

**Categories and subcategories**	**Example quotes from the data**^a^
***Themes of personal last wishes***	
**Travel**	
Vacation	“Make a round-the-world trip”, “Travel with my dog”
Special places/return to the place of birth	“Travel to see the Chinese wall”, “Go to Paris”, “One last visit to my place of birth – Hungary”, “To go back to Argentina”
To the ocean	“Drive along the Atlantic coast”, “Go to the North sea”
**Activities**	
Pleasant	“Dance on a music festival”, “Play the piano again”, “Take a long walk in the forest,”Cooking and eating sour herring”
Daring	“Jump off an airplane”,”Dive from a rock in Rio de Janeiro”
**Regaining Health**	
Overcome the disease	“Overcome cancer”, “I want to be healthy”
Live longer	“Have more days to live”,”To live longer, because I am still needed”
**Quality of life**	
Pain free	“Have no pain anymore and become more active”,”Be without pain, no suffering”
Mobility	“Be able to walk”,”To drive a car on my own”
Living as usual	“To be at home”, “To regain autonomy”,”To go back to work”,”Doing the usual things”
**Being with Family and Friends**	
Spend time together	“Spend time with the kids”, “Sitting together with friends smoking a cigarette”,”See my grandchildren again”,”To grow old with my sibling sister”
Share unique moments	“See my oldest son getting married”,”Christmas with my family”,”If there’s mercy I will celebrate my 70^th^ birthday with my kids”
Other aspects	“Be sure that my family will be okay”,”Saying goodbye to my brother, who I miss very much”
**Dying comfortably**	“Die at home”, “Die peacefully and without pain”, “Decide about when I die”,”Dying during sleep”
**Turn back time**	“Turn back time, being forty again”, “Be young again”, “I wish I had a time machine and could go back in time”
**Taking care of final matters**	“That the kids will make peace with each other”, “Get in touch with my daughter again”,”Being debt free”
***Meaning of personal last wishes***	
**Desiring fulfillment**	“I began to write down the story of my life…to leave a legacy”, “To have an electric wheelchair”
**Voicing hopes, dreams/fantasies**	“I hope to see my wife in paradise”, “I’ve always dreamt to stroke leopards”,”I am waiting for the miracle to be healthy”,”Hope for a cure”

^a^ The presented sentences are original quotes of patients (written free-text responses)

**Table 3:** Presence of a personal last wish and themes of personal last wishes at onset of specialist palliative care (cross-sectional analysis)

	**Entire sample** **(*N* = 361)**		**Home-based SPC** **(*N* = 238)**		**Inpatient SPC** **(*N* = 123)**		
	**n**	**%**	**n**	**%**	**n**	**%**	**p**
**Presence of a personal wish (yes)**	243	67.3	147	61.8	96	78.0	**.002**
**If yes, in the category of… **^**a**^	*N* = 243		*N* = 147		*N* = 96		
Travel	75	30.9	49	33.3	26	27.1	.303
Activities	43	17.7	28	19.0	15	15.7	.494
Regaining health	40	16.5	20	13.6	20	20.8	.137
Quality of life	37	15.2	22	14.3	15	15.6	.889
Being with family and friends	35	14.4	21	14.3	14	14.6	.948
Dying comfortably	14	5.8	8	5.4	6	6.3	.792
Turn back time	6	2.5	4	2.7	2	2.1	1.000^b^
Taking care of final matters	4	1.6	3	2.0	1	1.0	1.000^b^

*Abbreviations*: *p* probability of type I error (chi-square test / Fisher’s exact test), *SPC* specialist palliative care

Significant results are marked in bold

^a^ Multiple answers possible; percentage of patients who mentioned at least one personal last wish referring to this category; ^b^ Fisher’s exact test

**Table 4:** Presence of a personal last wish and themes of personal last wishes at follow-up (cross-sectional analysis)

	**Entire sample** **(*N* = 130)**		**Home-based SPC** **(*N* = 100)**		**Inpatient SPC** **(*N* = 130)**		***p***
	**n**	**%**	**n**	**%**	**n**	**%**	
**Presence of a personal last wish (yes)**	77	40.8	56	56.0	21	70.0	.171
**If yes, in the category of… **^**a**^	*N* = 77		*N* = 56		*N* = 21		
Travel	30	39.0	21	37.5	9	42.9	.184
Activities	12	15.6	10	17.9	2	9.5	.304^b^
Regaining health	18	23.4	15	26.8	3	14.3	.200^b^
Quality of life	15	19.5	13	23.2	4	19.0	.477^b^
Being with Family and Friends	15	19.5	8	14.3	7	33.3	.063
Dying comfortably	2	2.6	1	1.8	1	4.8	**–-**^c^
Turn back time	0	0.0	0	0.0	0	0.0	**–-**^c^
Taking care of final matters	1	1.3	0	0.0	1	4.8	**–-**^c^

*Abbreviations* *p* probability of type I error (chi-square tests / Fisher’s exact test), *SPC* specialist palliative care

^a^ Multiple answers possible; percentage of patients, who mentioned at least one personal last wish referring to this category; ^b^ Fisher’s exact test; ^c^ Significance not tested due to lacking variation in sample data

**Table 5:** Intra-personal changes of the presence of a personal last wish and themes of personal last wishes from the onset of specialist palliative care until follow-up (longitudinal analysis)

	**Onset of SPC compared to follow-up** **(entire sample: *N* = 130)**								
	steadily reported^**a**^		newly reported^**b**^		no longer reported^**c**^		steadily not reported^**d**^		
	**n**	**%**	**n**	**%**	**n**	**%**	**n**	**%**	**p**
**Presence of a personal last wish (yes)**	53	46.5	17	14.9	22	19.3	22	19.3	.522
**If yes, in the category of (*N* = 53)**									
Travel	13	24.5	9	17.0	7	13.2	24	45.3	.804
Activities	6	11.3	4	7.5	5	9.4	38	71.7	1.000
Regaining health	5	9.4	7	13.2	8	15.1	33	62.3	1.000
Quality of life	2	3.8	11	10.8	3	5.7	37	69.8	.057
Being with family and friends	2	3.8	7	13.2	6	11.3	38	71.7	1.000
Dying comfortably	0	0.0	0	0.0	1	1.9	52	98.1	**–-**^**e**^
Turn back time	0	0.0	0	0.0	1	1.9	52	98.1	**–-**^**e**^
Taking care of final matters	0	0.0	1	1.9	0	0.0	52	98.1	**–-**^**e**^

*Abbreviations* *p* probability of type I error (exact McNemar test)

^a^ Reported at the onset of SPC (t_0_) and at follow-up (t_1_); ^b^ Not reported at t_0_ but at t_1_; ^c^ Reported at t_0_ but not at t_1_; ^d^ Neither reported at t_0_ nor at t_1_; ^e^ Significance not tested due to lacking variation in sample data

**Table 6:** Results of univariable logistic regression for the presence of a personal last wish at the onset of specialist palliative care

	**β**	**SE**	**OR (95% CI)**	**p**
Age	-.007	.009	.993 (.976–1.011)	.458
Female gender	-.184	.225	.832 (.535–1.292)	.412
Married/life partnership	-.058	.225	.934 (.607–1.467)	.796
Have children	-.139	.253	.583 (.530–1.429)	.538
Living alone	-.083	.239	.920 (.576–1.470)	.727
Religious confession	.057	.232	1.059 (.672–1.669)	.805
School education				
Low (≤ 9 years)	.711	.278	2.036 (1.181–3.510)	.**010**
High (12–13 years)	.555	.291	1.742 (.985–3.079)	.056
Non-malignant disease	-.253	.359	.777 (.384–1.570)	.482
Physical symptom count (0–21)	.182	.035	1.199 (1.120–1.284)	** < .001**
Advance directive – living will	-.308	.245	.735 (.454–1.189)	.210
Advance directive – healthcare proxy	.082	.233	1.085 (.688–1.713)	.725
Palliative care ward	.789	.255	2.201 (1.334–3.631)	**.002**
Distress level (DT, 0–10)	.012	.053	1.012 (.913–1.122)	.820
Anxiety symptom level (GAD-2, 0–6)	.153	.060	1.165 (1.037–1.310)	**.010**
Depressive symptom level (PHQ-2, 0–6)	.079	.058	1.082 (.966–1.211)	.172

Reference groups: dependent variable: not having a personal last wish; independent variables: female gender vs. male; married/life partnership vs. single/divorced/widowed; having a child vs. not; living alone vs. living in the same household or near the family; religious confession vs. not; low (secondary general school-leaving certificate or less) and high (university entrance qualification) vs. intermediate school-leaving certificate (10 years); non-malignant disease vs. malignant; living will vs. none; health proxy vs. none; palliative care ward vs. home-based specialist palliative care

*Abbreviations*: *ß* unstandardized regression coefficient, *SE* standard error, *OR* odds ratio for independent variables, *CI* 95% confidence interval, *p* probability of type I error

Significant results are marked in bold

**Table 7:** Results of multivariable logistic regression for the presence of personal wishes at the onset of specialist palliative care

	**β**	**SE**	**OR (95% CI)**	**p**
School education				
Low (≤ 9 years)	.567	.293	1.764 (.994–3.130)	.052
High (12–13 years)	.503	.310	1.653 (.900–3.035	.105
Physical symptom count (0–21)	.156	.039	1.168 (1.083–1.260)	** < .001**
Palliative care ward	.687	.270	1.987 (1.171–3.372)	**.011**
Anxiety symptom level (GAD-2, 0–6)	.069	.554	1.053 (.920–1.204)	.457

Multivariable binary logistic regression analysis: *N* = 349 of 361 possible patients; Nagelkerke’s pseudo *R*^2^ = .131

Reference groups: dependent variable: not having a personal last wish; independent variables: low (secondary general school-leaving certificate or less) and high (university entrance qualification) vs. intermediate school-leaving certificate; palliative care ward vs. home-based specialist palliative care

*Abbreviations* *ß* unstandardized regression coefficient, *SE* standard error, *OR* odds ratio for independent variables, *CI* 95% confidence interval, *p* probability of type I error

Significant results are marked in bold

## Data Availability

The data that support the findings of this study are available from the corresponding author (AU), but restrictions apply to the availability of these data. The data are not publicly available because it contains information that could compromise participant’s informed written consent. Thus, data are available from the authors upon reasonable request and with the permission of the data protection officer of the University Medical Center Hamburg-Eppendorf, Hamburg, Germany.

## Abbreviation

SPC: Specialist palliative care
